# Personality traits and risk level for substance use among undergraduate medical students at an Ecuadorian university

**DOI:** 10.1192/j.eurpsy.2026.11000

**Published:** 2026-07-31

**Authors:** D. Mera, K. Toapanta, X. Sanchez

**Affiliations:** Pontificia Universidad Catolica Del Ecuador, Quito, Ecuador

## Abstract

**Introduction:**

The use of psychoactive substances constitutes a major public health problem worldwide(1). In the university setting, students have higher consumption rates than the general population of the same age, influenced by factors such as academic stress, social pressure, and adaptation to new environments. Personality traits indicate that certain personality traits are associated with a higher risk of substance use(2)

**Objectives:**

This study examined the relationship between personality traits and risk level for substance use among undergraduate students at one of the largest private universities in Ecuador.

**Methods:**

A cross-sectional survey of 397 randomly selected undergraduate medical students assessed personality traits (Salamanca Questionnaire) and risky substance use (ASSIST) from a population of 1,780. Sociodemographic data were collected. Associations were analyzed using Spearman correlations and mixed-effects logistic regression.

**Results:**

The participants (median age: 21.19 years, 66% women) had the highest consumption rates of alcohol, tobacco, and cannabis (93.2%, 49.6%, and 30.5%, respectively). Regarding overall risk, more than half of the sample (54.4%) were at moderate risk of consumption according to ASSIST criteria. The most frequent personality traits were histrionic, anxious, and borderline (26.7%, 24.4%, and 14.4%, respectively). The analysis revealed that narcissistic, antisocial, and impulsive traits maintained a positive and statistically significant association with the overall risk of substance use. The narcissistic trait showed a positive and significant association (OR = 1.58), indicating that the higher the level of this trait (0-2, 3-4, and 5-6), the greater the likelihood of belonging to a higher risk category of consumption. The antisocial trait has the most pronounced association (OR = 2.38), with each level of this trait more than doubling the probability of having a higher level of general consumption risk.

**Conclusions:**

A distinctive personality profile was identified in medical students with a high presence of emotional dysregulation traits (histrionic, borderline, and anxious traits). High-risk consumption was concentrated exclusively in alcohol and tobacco. Reforms and interventions are needed to promote student well-being and quality of care. Future research should evaluate interventions, conduct better personality diagnoses, and adopt longitudinal designs.

**References:**

Panamericana de la Salud. (2023). Substance abuse. https://www.paho.org/es/temas/abuso-sustancias

Alonso, A. C. (2014). Study of normal personality traits with the Salamanca Questionnaire [Internet]. https://gredos.usal.es/bitstream/handle/10366/127314/DPPMMLHM_CalderoAlonsoA_RasgospersonalidadcuestionarioSalamanca.pdf?sequence=1&isAllowed=y

**Disclosure of Interest:**

None Declared

